# Socioeconomic status indicators, physical activity, and overweight/obesity in Brazilian children

**DOI:** 10.1016/j.rppede.2015.08.018

**Published:** 2016

**Authors:** Victor Keihan Rodrigues Matsudo, Gerson Luis de Moraes Ferrari, Timóteo Leandro Araújo, Luis Carlos Oliveira, Emily Mire, Tiago V Barreira, Catrine Tudor-Locke, Peter Katzmarzyk

**Affiliations:** a Centro de Estudos do Laboratório de Aptidão Física de São Caetano do Sul (CELAFISCS), São Caetano do Sul, SP, Brazil; b Centro de Atendimento e Apoio ao Adolescente do Departamento de Pediatria da Universidade Federal de São Paulo (Unifesp), São Paulo, SP, Brazil; c Pennington Biomedical Research Center, Baton Rouge, LA, United States; d Syracuse University, New York, United States

**Keywords:** Accelerometry, Obesity, Adiposity, Sedentary lifestyle, Child

## Abstract

**Objective:**

To analyze the associations between socioeconomic status (SES) indicators and physical activity and overweight/obesity in children.

**Methods:**

485 children wore accelerometers for 7 days. Variables included time in sedentary behavior and moderate-to-vigorous physical activity (MVPA), and steps/day. Children were further categorized as meeting or not meeting guidelines of ≥60min/day MVPA and ≥12,000 steps/day. Body mass index (BMI) and body fat percentage (BF%) were measured using bioelectrical impedance. Overweight/obesity was defined as BMI >+1 SD and BF% ≥85th percentile. Parents answered questionnaires that questioned total annual household income, parental education level, parental employment status and automobile ownership.

**Results:**

Children averaged 59.5min/day in MVPA (44.1% met MVPA guidelines), and 9639 steps/day (18.4% met steps/day guidelines). 45.4% and 33% were overweight/obese classified by BMI and BF% respectively. Higher relative total annual household income level (*Odds Ratio* 0.31; 95% confidence interval=0.15-0.65), and relatively higher maternal (*OR*=0.38; 95%CI=0.20-0.72) and paternal (*OR*=0.36; 95%CI=0.17-0.75) education levels were associated with lower odds of children meeting MVPA guidelines. Household automobile ownership was associated with lower odds of children meeting MVPA (*OR*=0.48; 95%CI=0.31-0.75) and steps/day guidelines (*OR*=0.44; 95%CI=0.26-0.74).

**Conclusions:**

SES indicators were not associated with overweight/obesity, but higher SES was associated with lower odds of children meeting MVPA guidelines.

## Resumo

**Objetivo:**

Analisar as associações entre indicadores de nível socioeconômico (NSE) e atividade física e sobrepeso/obesidade em crianças.

**Métodos:**

485 crianças usaram acelerômetros por 7 dias. As variáveis incluíram o tempo em comportamentos sedentários e atividade física moderada a vigorosa (AFMV), e passos/dia. As crianças foram ainda classificadas como satisfazendo ou não o cumprimento das diretrizes de ≥60 min/dia MVPA e ≥12.000 passos/dia. Índice de massa corporal (IMC) e percentual de gordura corporal (%GC) foram medidos através de impedância bioelétrica. Sobrepeso/ obesidade foi definido como IMC>+1SD e um %GC≥percentil 85. Os pais responderam a questionários que questionavam o rendimento total anual das famílias, o nível de educação dos pais, situação de emprego dos pais e propriedade de automóvel.

**Resultados:**

As crianças mostraram uma média de 59,5 min/dia de AFMV (44,1% atingiram as diretrizes de AFVM), e 9.639 passos/dia (18,4% atingiram as diretrizes de passos/dia). 45,4% e 33% estavam com sobrepeso/obesidade classificada pelo IMC e %GC, respectivamente. Maior nível de renda familiar anual total (*odds ratio* 0,31; intervalo de confiança de 95%=0,15-0,65),e níveis relativamente mais elevados de educação materna (*OR*=0,38; IC95%=0,20-0,72) e paterno (*OR*=0,36; IC95%=0.17-0.75), foram associados com menor chance de crianças atingirem as diretrizes de AFVM. Propriedade de automóvel foi associada com menor chance de crianças atingirem as diretrizes de AFVM (*OR*=0,48; IC95%=0,31-0,75) e diretrizes de passos/dia (*OR*=0,44; IC95%=0,26-0,74).

**Conclusões:**

Os indicadores de NSE não foram associados com sobrepeso / obesidade, mas maior NES foi associado com menor chance de crianças atingirem diretrizes de AFVM.

## Introduction

Regular physical activity is associated with an array of health benefits for children.^1^ Unfortunately, the majority of children in Brazil are not sufficiently active. Currently, only 38.6% of boys and 20.1% of girls accumulate the recommended ≥60min/day of moderate-to-vigorous physical activity (MVPA).^2^ Further, the 2009 National Survey of School Health or Brazilian National Survey of School Health (Pesquisa Nacional de Saúde do Escolar - PeNSE) reported that one in three (33.5%) Brazilian children had overweight, and 16.6% of boys and 11.8% of girls were obese.^3^

Physical activity and overweight/obesity are influenced by complex factors that vary widely between countries.^4^^,^ 5 Socioeconomic status (SES) is one of these factors because it influences people's attitudes, experiences, and exposure to several health risk factors.^2^^,^ 6 Indeed, SES indicators (e.g., annual household income, parental education level and parental employment status) are related to a variety of chronic diseases in children.^7^^,^ 8 Although it has been differentially defined and measured, SES generally displays an inverse relationship with childhood overweight/obesity in developed countries.^5^^,^ 9

For example, in the United States, the Early Childhood Longitudinal Study-Birth Cohort (ECLS-B) showed that SES (defined as total annual household income and maternal education) was inversely associated with overweight/obesity [body mass index (BMI)>2 SD *z*-scores].^9^

Children's lack of physical activity and the high prevalence of overweight/obesity remain a combined challenge in developed countries, and now pose a growing public health threat.^10^ Paradoxically, although 80% of the global population is located in developing countries, only a small fraction of research focused on determinants of overweight/obesity and physical activity is conducted in these nations.^4^^,^ 11 A Kenyan study reported negative associations between SES indicators (defined as total household annual income, parental education level and public versus private school) and children's accelerometry, determined achievement of MVPA guidelines (defined and a mean of ≥60min/day at ≥3000 counts/min).^12^ In Brazil, however, Rezende et al.^2^ reported a significant positive association between maternal education level and self-reported physical activity in adolescents.

Given these polar findings, more research is required to understand the relationship between indicators of SES and adequate physical activity and overweight/obesity in children of developing countries. Further, only a few studies have used direct measures of physical activity and overweight/obesity, such as accelerometers or bioelectrical impedance, to examine associations with indicators of SES. Thus, the purpose of this study was to analyze the associations between parent-reported indicators of SES (annual household income, parental education level, parental employment status, and automobile ownership), child's enrollment at public versus private school, and direct measures of physical activity and overweight/obesity in Brazilian children.

## Method

Data were collected as part of the International Study of Childhood Obesity, Lifestyle and Environment (ISCOLE). The primary aim of ISCOLE was to investigate the influence of the physical, social, and policy environments on the relationship between children's lifestyle characteristics and obesity in 12 countries from all major world regions. Details of the ISCOLE study protocol are provided elsewhere.^13^ Data collection for this paper was conducted in the urban Brazilian city of São Caetano do Sul.

A random cluster sample of 564 5th grade Brazilian children (277 boys and 287 girls; 9-11 years of age) was assessed from 20 schools. After exclusion criteria (non-valid accelerometer data, details below), the final sample comprised 485 children (238 boys and 247 girls). Data were collected during the school year from March 2012 to April 2013. All assessments were conducted during a full week at each school.

Random lists of public (16) and private (4) elementary schools in the region of São Caetano do Sul, São Paulo, Southeast Brazil, were generated, and schools were selected from each list at a ratio of 4 (public) to 1 (private). This 80% public to 20% private schools ratio was purposely implemented to maximize SES distribution. If a school refused to participate in the study, it was replaced by the next school on the list, maintaining the same public to private school ratio. A random sample of 25-30 children was selected per school with a stipulation that each sex comprise 50% of the selected sample.

As described previously in detail,^13^ all data collection and management activities were performed and monitored locally following rigorous quality control procedures that were overseen by the ISCOLE Coordinating Center. Prior to participation, children and at least one of their parents/legal guardians were asked to sign the Instrument of Consent according to Resolution 196/96 of Brazil's National Health Council. Ethical approval was obtained from the Pennington Biomedical Research Center Institutional Review Board and the Federal University of São Paulo, Brazil.

One parent or legal guardian was asked to complete a questionnaire that included questions related to the health history of the child, total annual household income, parental education level, parental employment status and household automobile ownership. Many questions were asked separately about both maternal and paternal parents, but the identity of the familial relationship of the individual completing the questionnaire (mother or father) was not captured by the questionnaire. Total annual household income was used as a primary marker of family-level SES and was categorized into four categories based on the distribution of the data. These categories represent increasing levels of annual income (Brazilian currency) such that those with the lowest income were organized into the first category, and those with the highest income were in the last, as follows: <R$19,620 (level 1); R$19,621 to R$32,700 (level 2); R$32,701 to R$58,860 (level 3), and >R$58,861 (level 4). Additional indicators directly related to SES included parental education level (some high school or less, high school diploma/some college, or Bachelor's/professional degree), maternal and paternal employment status (none, less than part time, part time, or full time) and household automobile ownership (yes or no). Type of school, public versus private, as established during sampling was also considered an indicator of lower and higher SES, respectively.

New technologies applied to the measurement of body movement have emerged as an alternative method for assessment of physical activity. Instruments such as accelerometers provide new ways to estimate the frequency, duration, and intensity of physical activity in free-living individuals.^11^ Importantly, these methods avoid some of the inherent limitations of self-report instruments, that is, the recall bias. Accelerometry has become more common in studies of childhood physical activity around the world.^11^ Therefore, Actigraph GT3X+ accelerometers (ActiGraph, Ft. Walton Beach, United States) were used to objectively monitor sedentary behavior (SB), light physical activity (LPA), moderate physical activity (MPA), vigorous physical activity (VPA), MVPA and steps/day. The accelerometer was worn at the waist on an elasticized belt aligned with the mid-axillary line. The children were encouraged to wear the accelerometer 24h/day for at least 7 days (plus an initial familiarization day and the morning of the final day), including 2 weekend days. To be included in this analysis, children were required to have valid accelerometer data, defined as having ≥4 days (including at least one weekend day) with ≥10 hours of wake wear time per day.^14^^,^ 15

Data were collected at a sampling rate of 80Hz, downloaded in 1-s epochs, and re-integrated to 15s epochs.^16^ Activity count cut-points established by Evenson et al.^16^ for 15s epochs were applied to the data. The cut-points capture the sporadic nature of children's activity and provide the best classification accuracy among the currently available cut-points for physical activity in children.^14^ SB was defined as time accumulated at ≤25 activity counts/15s, >25 activity counts/15s for LPA, ≥574-1002 activity counts/15s for MPA, ≥1003 activity counts/15s for VPA, and ≥574 activity counts/15s for MVPA. Children were categorized as meeting (mean ≥60min/day) or not meeting MVPA guidelines,^17^ and meeting (mean ≥12,000 steps/day) or not meeting steps/day guidelines.^18^

Height, weight, BMI and body fat percentage (BF%) measures were obtained according to standardized ISCOLE procedures.^13^ Height was measured without shoes using a Seca 213 portable stadiometer (Hamburg, Germany), with the participant's head in the Frankfort Plane. Weight and BF% were measured using a portable Tanita SC-240 body composition analyzer (Arlington Heights, IL) after all outer clothing, heavy pocket items and shoes and socks were removed.^19^ The children were encouraged to come to the school following a 10h fast. There were no instructions provided to the participants regarding exercise the day before the test.^19^ Each child was measured twice and, when necessary, a third measurement was taken if the difference between the previous two were outside the permissible range for each measure and its replica (0.5cm for height and 0.5kg for weight). The mean value of each measured variable was used for analysis.

BMI was calculated using the standard formula weight (kg)/height (m)^2^ and, thereafter, BMI *z*-scores calculated based on growth reference data from the World Health Organization (WHO), and participants were further categorized as underweight: <−2 SD; normal weight: −2 SD to 1 SD; overweight: >+1 SD to 2; and obese: >+2 SD.^20^ BF% cut-points were calculated according to sex, and we defined those participants as overweight/obese whose BF% was ≥85th percentile of sex-specific reference data from children in the United Kingdom,^21^ since no other more population-appropriate reference data were available.

Descriptive statistics included means and standard deviations or frequencies, as appropriate. Multi-level logistic regression was used to determine the SES indicators that best predicted MVPA (mean ≥60min/day), steps/day (mean ≥12,000 steps/day), and overweight/obesity (BMI and BF%). Our set of models were run individually for each SES variable and allowed for clustering at the school level. We showed that only univariate models were employed due to issues of multi-collinearity among the SES indicator predictor variables.

The adjusted *Odds Ratio* (*OR*) and respective confidence interval (95%CI) were obtained from multivariable logistic regression models investigating the odds of four different outcomes: meeting MVPA guidelines,^17^ meeting steps/day guidelines,^18^ overweight/obese classification based on BF%,^21^ and overweight/obese classification based on BMI.^20^ A significance level of *p*<0.05 was used to interpret inferential analyses. All analyses were computed using Statistical Analysis System (SAS Institute, version 9.3, Cary, NC, United States).^22^

## Results

Characteristics of participating children and their parents are summarized in Tables 1 and 2. Of the 485 participants, 49% were boys and 51% were girls. Children's mean SB time was 499.7min/day (506.6min/day on weekdays versus 481.3min/day on weekend days). The mean daily time spent in LPA was 337.3min/day; 41.8min/day in MPA; 17.6min/day in VPA and 59.5min/day in combined MVPA. The children accumulated more MVPA (6.5min/day) on weekdays than weekend days. The percent of children who met MVPA guidelines was 44.1%. The children averaged 9639 steps/day, accumulated 752 more steps/day on weekdays than on weekend days and 18.4% of children met steps/day guidelines (Table 1).

**Table 1:** Descriptive analysis (mean [SD] or *n* [%]) of indicators of accelerometer-derived data and overweight/obesity of Brazilian children.

	Mean (SD) or *n* (%)
*Sex (n=485)*	
Boys	238 (49.0)
Girls	247 (51.0)
	
*SB and physical activity (n=485)*	
SB (min/day)	499.7 (69.05)
Weekday SB (min/day)	506.6 (74.8)
Weekend SB (min/day)	481.3 (98.3)
LPA (min/day)	337.3 (52.9)
MPA (min/day)	41.8 (16.6)
VPA (min/day)	17.6 (11.3)
MVPA (min/day)	59.5 (26.3)
Weekday MVPA (min/day)	61.4 (26.9)
Weekend MVPA (min/day)	54.9 (35.2)
Daily steps (steps/day)	9639 (2.780)
Weekday steps (steps/day)	9857 (2.846)
Weekend steps (steps/day)	9105 (3.982)
Meeting MVPA guidelines (mean ≥60min/day)^17^	214 (44.1%)
Meeting steps/day guidelines (≥12,000 steps/day)^18^	89 (18.4%)
BF% (*n*=485)	23.0 (9.2)
	
*BF% categories (United Kingdom reference data)* 21	
Normal	325 (67%)
Overweight/obesity	160 (33%)
BMI (kg/m^2^) (*n*=485)	19.8 (4.4)
	
*BMI categories (WHO cut-points)* 20	
Underweight	11 (2.3%)
Normal weight	254 (52.4%)
Overweight	112 (23.1%)
Obese	108 (22.3%)

SB, sedentary behavior; LPA, light physical activity; MPA, moderate physical activity; VPA, vigorous physical activity; MVPA, moderate/vigorous physical activity; min: minutes. BF%, body fat percentage; BMI, body mass index; WHO, World Health Organization.

**Table 2:** Descriptive analysis *n* (%) of indicators of SES of Brazilian children.

	Mean (SD) or *n* (%)
*Participation by type of school*	
Public	37 (7.6%)
Private	448 (92.4%)
	
*Total annual household income (n=383)*	
Level 1 (<R$19,620)	149 (38.9%)
Level 2 (19,621-<32,700)	101 (26.3%)
Level 3 (32,701-58,860)	81 (21.1%)
Level 4 (>R$ 58,861)	52 (13.6%)
	
*Maternal education level (n=441)*	
Some high school or less	155 (35.1%)
High school diploma/some college	216 (49.0%)
Bachelor's or professional degree	70 (15.9%)
	
*Paternal education level (n=430)*	
Some high school or less	177 (41.2%)
High school diploma/some college	197 (45.8%)
Bachelor's or professional degree	56 (13.0%)
	
*Maternal employment status (n=438)*	
None	101 (23.1%)
Less than part time	57 (13.0%)
Part time	67 (15.3%)
Full time	213 (48.6%)
	
*Paternal employment status (n=404)*	
None	40 (9.9%)
Less than part time	33 (8.2%)
Part time	66 (16.3%)
Full time	265 (65.6%)
	
*Automobile ownership (n=441)*	
No	137 (31.1%)
Yes	304 (68.9%)

R$, Brazilian Real.

The mean BF% was 23% and we found that 33% of children exceeded the applied BF% cut-point that represented the 85th percentile of the reference data.^21^ On the other hand, based on WHO BMI *z*-score categorization,^20^ we found that 2.3% of children were underweight, 52.4% were normal weight, 23.1% were overweight, and 22.3% were obese (a combined 45.4% were overweight/obese) (Table 1).

Of the all participants, 92.4% attended public schools, while 7.6% were enrolled in private schools. Of the 485 questionnaires distributed to children's parents/legal guardians, we received 383 with information about total annual household income, 441 with maternal education level, 430 with paternal education level (429 with both maternal and paternal education level), 438 with maternal employment status, 404 with paternal employment status (401 with both maternal and paternal employment status) and 441 with household automobile ownership information (Table 2).

A majority of reported total annual household income (38.9%) fell into the low category (level 1), while 26.3%, 21.1% and 13.6% comprised levels two through four, respectively. A higher proportion of mothers (15.9%) than fathers (13.0%) were reported as having attained a Bachelor's/professional degree. Responses to maternal and paternal employment status indicated that 48.6% and 65.6% worked full time, respectively. A higher proportion of mothers (23.1%) than fathers (9.9%) did not work and 68.9% of the children came from households with at least one automobile (Table 2).

Table 3 presents the results of the multi-level logistic regression analysis describing the association between the selected SES indicators and meeting MVPA and steps/day guidelines. These analyses were performed using multilevel models that allowed the intercept to vary for each school. As shown in Table 3, multi-level logistic regression analysis revealed that girls were at significantly lower odds of meeting MVPA guidelines when compared to boys.

**Table 3:** Multi-level logistic regression for indicators of SES with MVPA and steps/day guidelines in Brazilian children.

Variables	Meeting MVPA guidelines			Meeting steps/day guidelines	
	*OR* (95%CI)	*p* -value		*OR* (95%CI)	*p* -value
*Sex*		<0.0001			<0.0001
Boys	1			1	
Girls	0.16 (0.11-0.25)			0.28 (0.16-0.47)	
					
*Participation by type of school*		0.6890			0.3002
Public	1			1	
Private	1.22 (0.45-3.29)			1.61 (0.64-4.06)	
					
*Total annual household income*		0.0045			0.1283
Level 1 (<R$19,620)	1			1	
Level 2 (19,621-<32,700)	0.78 (0.46-1.32)			0.93 (0.50-1.73)	
Level 3 (32,701-58,860)	0.45 (0.25-0.81)			0.46 (0.21-1.02)	
Level 4 (>R$ 58,861)	0.31 (0.15-0.65)			0.45 (0.17-1.18)	
					
*Maternal education level*		0.0092			0.4743
Some high school or less	1			1	
High school diploma/some college	0.66 (0.43-1.02)			0.71 (0.41-1.22)	
Bachelor's or professional degree	0.38 (0.20-0.72)			0.84 (0.40-1.80)	
					
*Paternal education level*		0.0232			0.2429
Some high school or less	1			1	
High school diploma/some college	0.88 (0.58-1.34)			0.99 (0.58-1.66)	
Bachelor's or professional degree	0.36 (0.17-0.75)			0.43 (0.15-1.19)	
					
*Maternal employment status*		0.1623			0.5965
None	1			1	
Less than part time	1.24 (0.62-2.49)			0.57 (0.22-1.48)	
Part time	1.47 (0.75-2.86)			0.83 (0.36-1.92)	
Full time	1.79 (1.06-3.02)			1.02 (0.55-1.90)	
					
*Paternal employment status*		0.0623			0.4524
None	1			1	
Less than part time	0.44 (0.17-1.18)			0.89 (0.21-3.69)	
Part time	0.36 (0.15-0.84)			2.00 (0.65-6.11)	
Full time	0.38 (0.19-0.78)			1.58 (0.58-4.3)	
					
*Automobile ownership*		0.0011			0.0021
No	1			1	
Yes	0.48 (0.31-0.75)			0.44 (0.26-0.74)	

There were negative associations between total annual household income, both maternal and paternal education level and meeting MVPA guidelines. Higher total household annual income and higher levels for both parents education were associated with lower odds of the child meeting MVPA guidelines. Children who came from households with at least one automobile were also less likely to meet MVPA guidelines. On the other hand, there were no significant associations between type of school, either maternal or paternal employment status, and meeting MVPA guidelines.

Girls and children from households with at least one automobile were less likely to meet steps/day guidelines. Meeting steps/day guidelines was not associated with type of school, total annual household income, maternal or paternal education levels or employment status.

Table 4 presents the multi-level logistic regression analysis for overweight/obesity, accounting for school-level clustering. There were no significant associations between any SES indicator and overweight/obesity as defined by BF% or BMI in Brazilian children.

**Table 4:** Multi-level logistic regression for indicators of SES with BF% and BMI overweight/obesity in Brazilian children.

Variables	BF% overweight/obese			BMI overweight/obese	
	*OR* (95%CI)	*p* -value		*OR* (95%CI)	*p* -value
*Sex*		0.5471			0.6208
Boys	1			1	
Girls	0.88 (0.60-1.31)			0.91 (0.63-1.32)	
					
*Participation by type of school*		0.3266			0.9396
Public	1			1	
Private	0.61 (0.23-1.66)			0.97 (0.39-2.37)	
					
*Total annual household income*		0.6269			0.6571
Level 1 (<R$19,620)	1			1	
Level 2 (19,621-<32,700)	1.44 (0.83-2.50)			1.39 (0.82-2.35)	
Level 3 (32,701-58,860)	1.17 (0.64-2.13)			1.18 (0.67-2.09)	
Level 4 (>R$58,861)	1.20 (0.59-2.43)			1.07 (0.55-2.09)	
					
*Maternal education level*		0.4782			0.6013
Some high school or less	1			1	
High school diploma/some college	0.80 (0.51-1.26)			0.99 (0.64-1.52)	
Bachelor's or professional degree	0.70 (0.37-1.32)			0.75 (0.41-1.37)	
					
*Paternal education level*		0.2272			0.5330
Some high school or less	1			1	
High school diploma/some college	0.77 (0.49-1.19)			0.88 (0.57-1.33)	
Bachelor's or professional degree	0.56 (0.27-1.14)			0.69 (0.57-1.33)	
					
*Maternal employment status*		0.8611			0.8895
None	1			1	
Less than part time	0.98 (0.47-2.00)			0.82 (0.42-1.62)	
Part time	1.26 (0.64-2.50)			1.06 (0.55-2.02)	
Full time	1.17 (0.68-1.99)			0.90 (0.54-1.48)	
					
*Paternal employment status*		0.1012			0.2434
None	1			1	
Less than part time	0.70 (0.23-2.12)			0.91 (0.35-2.38)	
Part time	2.09 (0.88-4.97)			1.81 (0.80-4.08)	
Full time	1.20 (0.57-2.54)			1.06 (0.53-2.10)	
					
*Automobile ownership*		0.0699			0.2074
No	1			1	
Yes	1.53 (0.96-2.45)			1.31 (0.85-2.01)	

## Discussion

The aim of this study was to examine the association between indicators of SES, physical activity and overweight/obesity in a sample of Brazilian children. Female gender was negatively associated with meeting MVPA guidelines. Total annual household income, parental education level (both maternal and paternal), and household automobile ownership were all negatively associated with children meeting MVPA guidelines when adjusted for school and sex. Household automobile ownership was also negatively associated with children meeting steps/day guidelines. On the other hand, we found no significant association between any SES indicator and overweight/obesity in these Brazilian children.

Several surveys conducted in developed countries such as United States,^7^ France^23^ found no significant association between accelerometry-determined achievement of MVPA and SES indicators (total annual household income and maternal education level). On the other hand, the findings of this study showed that higher total annual household income and parental education level was associated with children spending less time in MVPA, pointing to a negative household SES relationship with children's MVPA, which supports previous research that has shown a negative relationship between indicators of SES and children's MVPA in developing countries.^12^ For example, Muthuri et al.^12^ found higher total annual household income was associated with lower MVPA in Kenyan children 9-11 year of age. However, their definition of MVPA (defined as≥3000 counts/min) and total annual household income (low, medium and high) differed from our own. Also different from the results of our study (*OR*=1.22; 95%CI=0.45-3.29), the authors found children attending private schools had a 96% lower odds of being active when compared to those attending public school.

Due to the logistical and financial challenges associated with accelerometry research, Brazilian researchers have previously relied on questionnaires to quantify physical activity behaviors.^2^^,^ 24 Rezende et al.^2^ analyzed self-reported data from the nationally representative Brazil National Adolescent School-based Health Survey (Pesquisa Nacional de Saúde do Escolar - PeNSE), in 2012, and reported a significant and positive association between indicators of SES (mother's education level and type of school) and physical activity (defined as reporting >300min/week) of Brazilian adolescents 13-16 years. In contrast to our study, the authors found that adolescents of mothers who completed high school diploma/some college and Bachelor's/professional degree education had a 20% higher prevalence ratio of being classified as physically active when compared with adolescents with mothers who did not complete some high school or less. Further, adolescents attending public schools had a 14% lower odds of being classified as physically active compared to adolescents attending private schools. The authors did not find a significant association between household automobile ownership and adolescent's physical activity. Hallal et al.^24^ found that Brazilian children (10-12 years age) attending private schools had a 16% higher risk ratio of reporting being physically inactive (defined as <300min/week) compared to children in public schools. Hallal et al.^24^ showed a negative association between family SES and children's physical inactivity. Children at the highest level of SES had a 27% higher risk ratio of being physically inactive when compared with the lowest SES level.^24^

The proportion of children meeting the physical activity guidelines in Brazil (44.1%) is higher than some Western countries such as the United States, where objective measures revealed that only 29% of children and youth accumulated the recommended ≥60min/day MVPA.^25^ In developing countries, only 12.6% (Kenya)^12^ and <1% (South Africa)^26^ of children met MVPA guidelines, however, the criteria for meeting MVPA guidelines differs across studies.

In this sample of children from São Caetano do Sul, São Paulo, Southeast Brazil, mean daily time spent in SB was 499.7min/day, LPA was 337.3min/day, and MVPA was 59.5min/day. Contrasting these results with data from children and adolescents with mean age of 13.3 years from another region (Pelotas, Southern Brazil), Hallal et al.^27^ reported higher accelerometer-determined SB (660min/day) and lower values for LPA (189min/day) and MVPA (35min/day). Their definition of SB (defined as ≤100 counts/min), LPA (defined as >100-2295 counts/min) and MVPA (defined as ≥2296 activity counts/min) differed from our own (SB defined as ≤25 activity counts/15s; LPA defined as >25 activity counts/15s and MVPA defined as ≥574 activity counts/min).

Also in contrast to our findings, Drenowatz et al.^7^ divided a sample of American children 8-11 years of age into five groups by total annual household income (<R$24,999; R$25,000 to R$35,999; R$36,000 to R$54,999; R$55,000 to R$100,000; >R$100,000) and used pedometers to evaluate steps/day. The authors found a positive association between steps/day and annual household income. The group with higher total annual household income (12,270 steps/day) walked 1335 steps/day more than the group with the lowest total annual household income (10,935 steps/day). We showed no association between total annual household income and meeting steps/day guidelines.

The results revealed substantial proportions of childhood overweight/obesity (45.4%) for this age group. These figures are higher than values in developed countries such as in the United States and Canada, which have childhood overweight/obesity (BMI defined as ≥85th percentile) prevalence of 33% and 29%, respectively.^28^^,^ 29 These findings are indicative of an existing threat of childhood overweight/obesity among Brazilian's school-aged children from in the city of São Caetano do Sul.

In Brazil, recent data have also revealed high prevalence of overweight/obesity in children. The results indicate that 37.2% Brazilian children of 10-11 years of age were overweight/obese (defined as >+1 SD).^3^ In the present study, we found a similarly high (45.8% BMI) prevalence of overweight/obesity in one region.

Contrasting our results regarding the SES indicators, the *National Health and Nutrition Examination Survey* (NHANES) data from the United States found a significant inverse association between parental education level and total annual household income with overweight/obesity (BMI defined as ≥85th percentile) in children.^30^ We did not find that any SES indicator defined herein was associated with children's' overweight/obesity.

An obvious limitation to this research is our cross-sectional study design that provided no basis for studying causality. Further, even though the study involved schools from São Caetano do Sul, it does not provide a nationally representative sample. Nevertheless, this study provides robust objective measures of several behavioral risk factors and related correlates among a large group of school children. It provides evidence for a high prevalence of physical inactivity and overweight/obesity in children from the city of São Caetano do Sul, Brazil. In contrast to findings from developed countries, we found that higher total annual household income and parental education level were associated with a lower likelihood of children meeting MVPA guidelines. Household automobile ownership was negatively associated with meeting both the MVPA and steps/day guidelines. We did not find any association between any SES indicators and overweight/obesity in this age restricted sample.

Longitudinal studies are needed to provide a better understanding of the causal relationship between SES indicators, MVPA, steps/day and overweight/obesity, during childhood which could contribute to a higher success-rate of interventions. It is important to develop broad national policies and programs to fight the physical inactivity and obesity epidemic.
